# Supplementary material for: Forty-five patient-derived xenografts capture the clinical and biological heterogeneity of Wilms tumor
Source: Nat Commun. 2019 Dec 20;10:5806. doi: 10.1038/s41467-019-13646-9 (PMC6925259; doi:10.1038/s41467-019-13646-9)
Supplement: Supplementary file 1 — Supplementary Information File [file 41467_2019_13646_MOESM1_ESM.pdf]

Supplementary Information

Forty-five patient-derived xenografts capture the clinical and biological heterogeneity of Wilms tumor

Andrew J Murphy[1,2,*], Xiang Chen[3], Emilia M Pinto[4], Justin Williams[3], Michael R Clay[4], Stanley B Pounds[5], Xueyuan Cao[5,6], Lei Shi[5], Tong Lin[5], Geoffrey Neale[7], Christopher L Morton[1], Mary A Woolard[1], Heather L Mulder, Hyea Jin Gil[1], Jerold E Rehg[4], Catherine A Billups[5], Matthew L Harlow[8], Jeffrey S Dome[9], Peter J Houghton[10], John Easton[3], Jinghui Zhang[3], Rani E George[8], Gerard P Zambetti[4], Andrew M Davidoff[1,2]

[1]Department of Surgery, St. Jude Children's Research Hospital, 262 Danny Thomas Place, Memphis, TN 38105, USA

[2]Division of Pediatric Surgery, Department of Surgery, University of Tennessee Health Science Center, 910 Madison Ave. 2nd floor, Memphis, TN 38163, USA

[3]Department of Computational Biology, St. Jude Children's Research Hospital, 262 Danny Thomas Place, Memphis, TN 38105, USA

[4]Department of Pathology, St. Jude Children's Research Hospital, 262 Danny Thomas Place Memphis, TN 38105, USA

[5]Department of Biostatistics, St. Jude Children's Research Hospital, 262 Danny Thomas Place, Memphis, TN 38105, USA

[6]College of Nursing, University of Tennessee Health Science Center, 920 Madison Ave, Memphis, TN 38163, USA

[7]Hartwell Center for Bioinformatics and Biotechnology, St. Jude Children's Research Hospital, 262 Danny Thomas Place, Memphis, TN 38105, USA

[8]Department of Pediatric Hematology and Oncology, Dana-Farber Cancer Institute and Boston Children's Hospital, Harvard Medical School, 450 Brookline Avenue, Room D640E, Boston, MA 02215, USA

[9]Division of Oncology, Children's National Medical Center, 111 Michigan Avenue NW, Washington DC 20010, USA

[10]Greehey Children's Cancer Research Institute, University of Texas Health Science Center,

8403 Floyd Curl Drive, San Antonio, TX 78229, USA

Supplementary Methods

Supplementary Figures 1-10

Supplementary References

Supplementary Methods

Whole exome sequencing (WES). Genomic DNA was quantified using the Quant-iT RiboGreen assay (Life Technologies). Libraries for WES were prepared according to the Illumina TruSeq DNA Exome protocol (P/N 20020615). In brief, 100 ng DNA was sheared using a Covaris LE220 ultrasonicator, then blunt-end repaired and size-selected by bead purification prior to addition of dA-tails and ligation of Illumina sequencing adapters containing unique-dual indexes. Ligated fragments were amplified for 8 cycles and cleaned up by bead purification, then 100 ng of each library was hybridized within a 12-plex library pool to Coding Exome Oligos (45 Mb target design). Hybridization enrichment of the pooled libraries was performed twice according to the recommended conditions of the protocol. The enriched libraries were then amplified for 8 cycles, purified by bead clean up, and validated by fragment size analysis on a BioAnalyzer 2100 prior to sequencing. Enriched library insert sizes were an average of 150-200 bp. Sequencing was performed on an Illumina NovaSeq 6000 instrument using paired 100 cycle dual indexed chemistry. This protocol was followed for all sample types, including germline, primary tumor, and xenografts (indicated in sample IDs as G, D, or X, respectively).

WES mapping, coverage and quality assessment, single-nucleotide variant (SNV) and insertion/deletion detection, tier annotation for sequence mutations, and prediction of the deleterious effects of missense mutations was performed as previously described[1]. Single nucleotide and insertion/deletion variants were validated by targeted capture amplicon sequencing using the MiSeq platform (Illumina) and Validation Capture pipeline and/or Sanger sequencing. For WTPDX WES and targeted capture sequencing, the XenoCP method was used to remove murine reads misaligned to the human genome.

To illustrate the subclonal analysis, the mutant allele frequencies from the target capture sequencing dataset (mutant allele\total reads) for each paired primary tumor and xenograft were plotted against one another using the Python v2.7.2 software Seaborn package. Fish plots were used to illustrate the evolution of mutant allele frequencies between primary tumors and xenografts by manually selecting clones and plotting them using the R graphics package "fishplot" as previously described[2].

RNA-sequencing and gene expression microarray analysis. Total RNA was extracted from 37 paired primary tumors and WTPDX, 8 additional WTPDX without available primary tumor RNA, and 3 normal kidney specimens by using the Qiagen RNeasy Midi kit (Qiagen). Commercially available pooled total RNA from four human fetal kidney specimens was also included (Takara, Kusatsu, Japan). RNA was quantified using the Quant-iT RiboGreen assay (Life Technologies) and quality checked by 2100 Bioanalyzer RNA 6000 Nano assay (Agilent) or LabChip RNA Pico Sensitivity assay (PerkinElmer) prior to library generation. Libraries were prepared from 250 ng to 1000 ng of total RNA with the TruSeq Stranded Total RNA Library Prep Kit according to the manufacturer's instructions (Illumina P/N 20020613). Libraries were analyzed for insert size distribution on a 2100 BioAnalyzer High Sensitivity kit (Agilent Technologies) or Caliper LabChip GX DNA High Sensitivity Reagent Kit (PerkinElmer). Libraries were quantified using the Quant-iT PicoGreen ds DNA assay (Life Technologies) or low pass sequencing with a MiSeq nano kit (Illumina). Sequencing was performed on an Illumina NovaSeq 6000 instrument using paired 100 cycle dual indexed chemistry. This protocol was followed for

all sample types, including primary tumor and xenografts (indicated in sample IDs as D or X, respectively).

RNA reads were mapped as previously described. RNA-seq gene level read counts and FPKM were generated using HTseq-count using R software (www.r-project.org, Aukland, New Zealand), based on transcript models in GENCODE v19. All subsequent analyses were performed using Python v2.7.2 (using packages SciPy, Pandas, NumPy, Scikit-learn, and Seaborn). For all RNA-seq-based analyses, Fragments Per Kilobase Million (FPKM) values were transformed by log2(FPKM+0.01); all genes with max(log2(FPKM+0.01)) < 1 were excluded. A pairwise Spearman correlation matrix (SciPy spearmanr) heatmap was constructed to compare gene expression between primary tumors and corresponding WTPDX. The paired primary tumors and corresponding WTPDX were first collapsed by taking the mean Spearman correlation for each pairwise comparison (e.g. for n primary/PDX pairs, a [2*n* X 2*n*] correlation matrix was collapsed to a [*n* X *n*] matrix.). These collapsed pairs were clustered, then expanded to include the paired primary tumors and WTPDX in order. Hierarchical clustering was performed using SciPy.hierarchy.linkage (method="complete", metric="euclidean") and plotted using package Seaborn clustermap with the linkages computed from the SciPy package.

Mean log2(FPKM+0.01) values for each expressed gene were compared between primary tumors and WTPDX using a paired two-tailed *t*-test (SciPy.stats ttest_rel) with FDR 0.05 correction (statsmodels.stats.multitest fdrcorrection [method="fdr_bh"]). Gene list analysis was performed using lists from the the PANTHER v13.1 (Protein Analysis Through Evolutionary Relationships; http://www.pantherdb.org/, Los Angeles, CA) database to identify differences in gene pathway expression between primary tumors and WTPDX and statistical methods derived from the Enrichr gene list enrichment analysis tool[3,4]. Briefly, for gene list statistical analysis, the *p* value for enrichment was calculated using Fisher's exact test. The adjusted p value was calculated using the Benjamini-Hochberg method for correction for multiple hypotheses testing. The *z* score was computed using a modification to Fisher exact test in which a *z* score is computed for deviation from an expected rank. The combined score *c* was calculated by combining the *p* value and *z* score by multiplying the two scores as follows: $c = \ln(p) * z$. Gene list analysis was performed on the overall group of paired primary tumors and WTPDX and an additional subgroup analysis was performed on primary tumor-WTPDX pairs using the first quartile of Spearman correlation values as a threshold ($r<.836$) to explore differences in the cohort with most differential gene expression.

Principal component analysis (PCA) was applied to the entire dataset and paired primary tumor and WTPDX samples, normal kidney samples, and fetal kidney samples were plotted by the first two computed principal components (using sklearn.decomposition PCA). To further explore the resulting PCA clustering patterns, the contribution of previously described gene sets associated with cellular lineages in kidney development (cap mesenchyme, uninduced mesenchyme, kidney epithelium, podocytes) and WT histologic archetypes (blastemal, epithelial, and stromal archetypes) was assessed. To perform this analysis, the Spearman correlation statistic comparing the collapsed kidney developmental or WT archetype gene set (average log2(FKPM+0.01), standardized by removing the mean and scaling to unit variance) and each principle component was computed.

A z score normalized heatmap display of primary tumor and WTPDX gene expression of genes associated with the immune response (GO Biological Processes GO: 0006955 IMMUNE_RESPONSE), the VEGF pathway (Affymetrix gene set Neutrophilin interactions with VEGF and VEGFR), the metanephric mesenchyme (GO_METANEPHRIC_MESENCHYME_DEVELOPMENT), and genes previously shown to be upregulated in Wilms tumor versus normal and fetal kidney (LI_WILMS_TUMOR_VS_FETAL KIDNEY_2_UP) was generated. Clustering was performed using Seaborn clustermap (method="complete", metric="euclidean").

To validate RNA-seq findings using a separate assay, we also performed transcriptome-wide gene-level expression profiling in a selected group of 16 WTPDX, 13 available corresponding primary tumor specimens, 3 normal kidney specimens, and commercially available pooled RNA from four human fetal kidney specimens (Takara) using the Human Clariom S assay (ThermoFisher) as per the manufacturer's protocol and analyzed by Expression Console software (ThermoFisher). Expression microarray data were normalized by robust multi-array averages[5]. PCA was applied to the entire expression matrix. For the set of WTPDX-primary tumor pairs, the Spearman correlation of expression in the xenograft with expression in the primary tumor was computed for each probe-set. The p values were computed using Fisher's z transformation of the correlation statistic. The false discovery rate was computed using Storey's method, with the proportion of tests with a true null hypothesis estimated by twice the average p value[6].

Response and Event Definitions for Solid Tumor Xenograft Models[5,6] *Response:* For individual mice, progressive disease (PD) was defined as < 50% regression from initial volume during the study period and > 25% increase in initial volume at the end of study period. Stable disease (SD) was defined as < 50% regression from initial volume during the study period and ≤ 25% increase in initial volume at the end of the study. Partial response (PR) was defined as a tumor volume regression ≥50% for at least one time point but with measurable tumor (≥ 0.10 cm^3). Complete response (CR) was defined as a disappearance of measurable tumor mass (< 0.10 cm^3) for at least one time point. A complete response was considered maintained (MCR) if the tumor volume was <0.10 cm^3 at the end of the study period. For treatment groups only, if the tumor response was PD, then the PD was further classified into PD1 or PD2 based on the tumor growth delay value. TGD values were calculated based on the number of days to event. For each individual mouse that had PD and had an event in the treatment groups, a TGD value was calculated by dividing the median time to event for that mouse by the median time to event in the respective control group. Median times to event were estimated based on the Kaplan-Meier event-free survival distribution. If a mouse had a TGD value ≤ 1.5, that mouse was considered PD1. If the TGD value was > 1.5, the mouse was considered PD2. Mice that had PD but did not have an event at the end of the study were coded as PD2.

Event-free survival: An event in the solid tumor xenograft models was defined as a quadrupling of tumor volume from the initial tumor volume. Event-free survival was defined as the time interval from initiation of study to the first event or to the end of the study period for tumors that did not quadruple in volume. The time to event was determined using interpolation based on the formula: $t_x = t_1 + (t_2 - t_1) ln (V_e / V_1) / ln(V_2 / V_1)$, where t_x is the interpolated day to event, t_1 is the lower observation day bracketing the event, t_2 is the upper observation day bracketing the event, V_1 is the tumor volume on

day t_1 , V_2 is the tumor volume on day t_2 and V_e is the event threshold (4 times initial tumor volume for solid tumor xenografts).

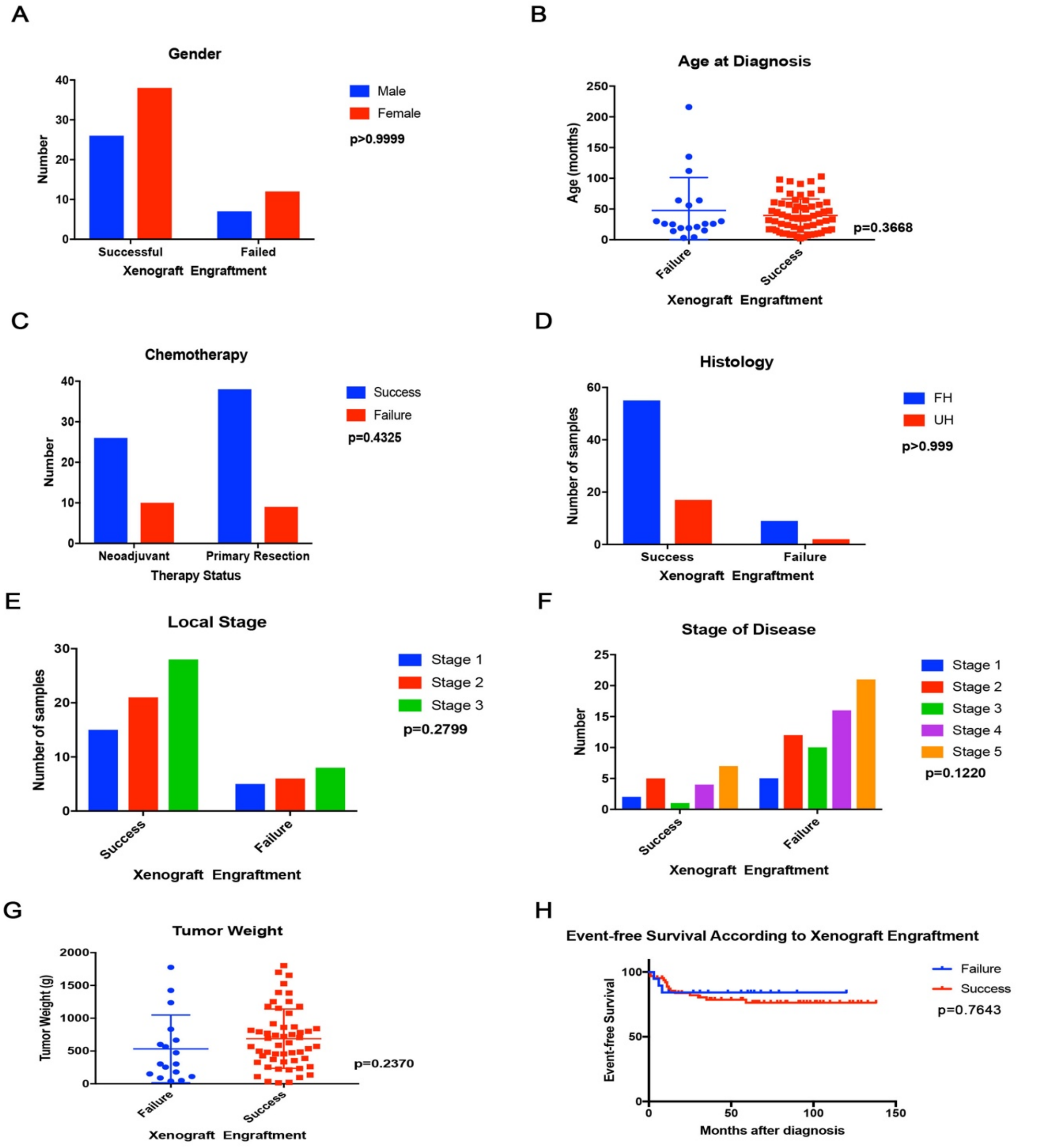

Supplementary Figure 1. Clinical characteristics were not associated with the success of xenograft engraftment. No significant differences were found between primary tumors for which xenograft engraftment was successful or failed with respect to (A) patient gender (Fisher exact test $p>0.9999$), (B) age at diagnosis (unpaired two-tailed *t* test p=0.3668; line is mean with tails showing standard deviation), (C) primary resection or neoadjuvant chemotherapy status (Fisher exact test $p=0.4325$), (D) histology (Fisher exact test $p>0.999$), (E) local stage (two-way ANOVA $p=0.2799$), (F) disease stage (two-way ANOVA $p=0.1220$), (G) primary tumor weight (unpaired two-tailed *t*-test p=0.2370; line is mean with tails showing standard deviation), or (H) event-free survival (Gehan-Breslow-Wilcoxon test $p=0.7643$).

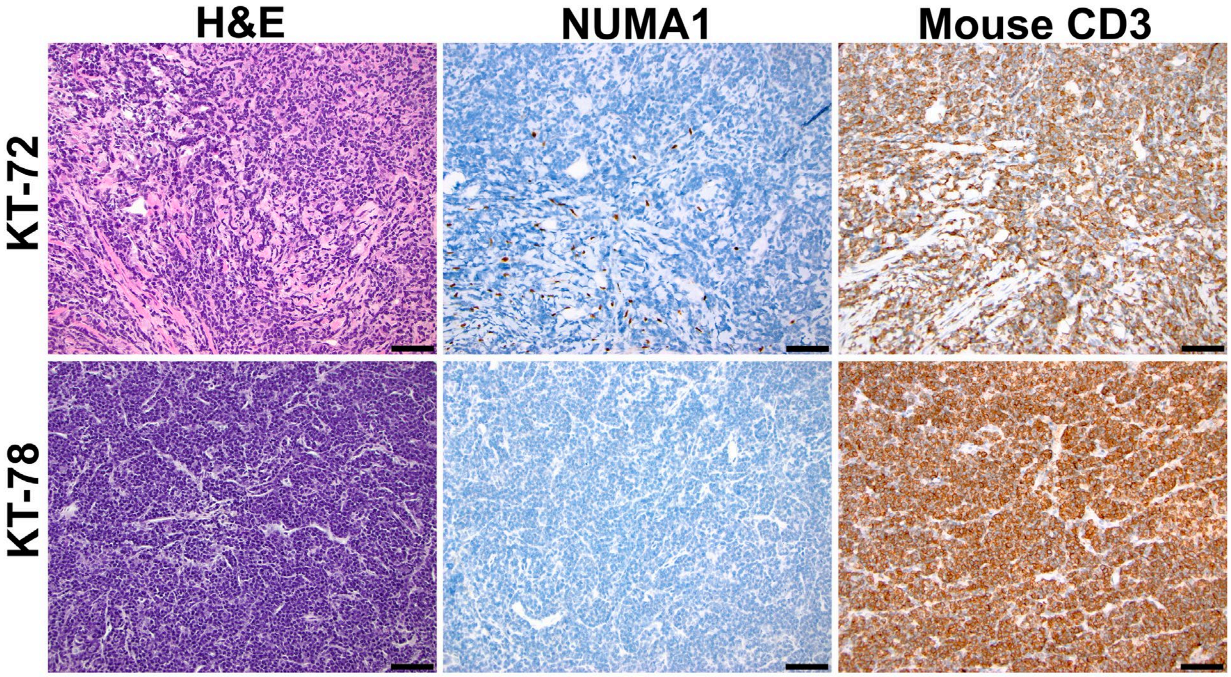

Supplementary Figure 2. Two WTPDX samples were consistent with murine T-cells. KT-72 (top panels) and KT-78 (bottom panels) were excluded from this study because STR DNA profiling could not amplify human DNA. Hematoxylin and eosin stained sections (left panels) revealed small round blue cell morphology. Human cell-specific nuclear antigen 1 staining (NUMA1, middle panels) demonstrated only scant human cells for KT-72 and no human cells for KT-78. Murine CD3 immunohistochemistry (right panels) demonstrated both KT-72 and KT-78 were consistent with murine T-cells. Scale bar = 100 µm.

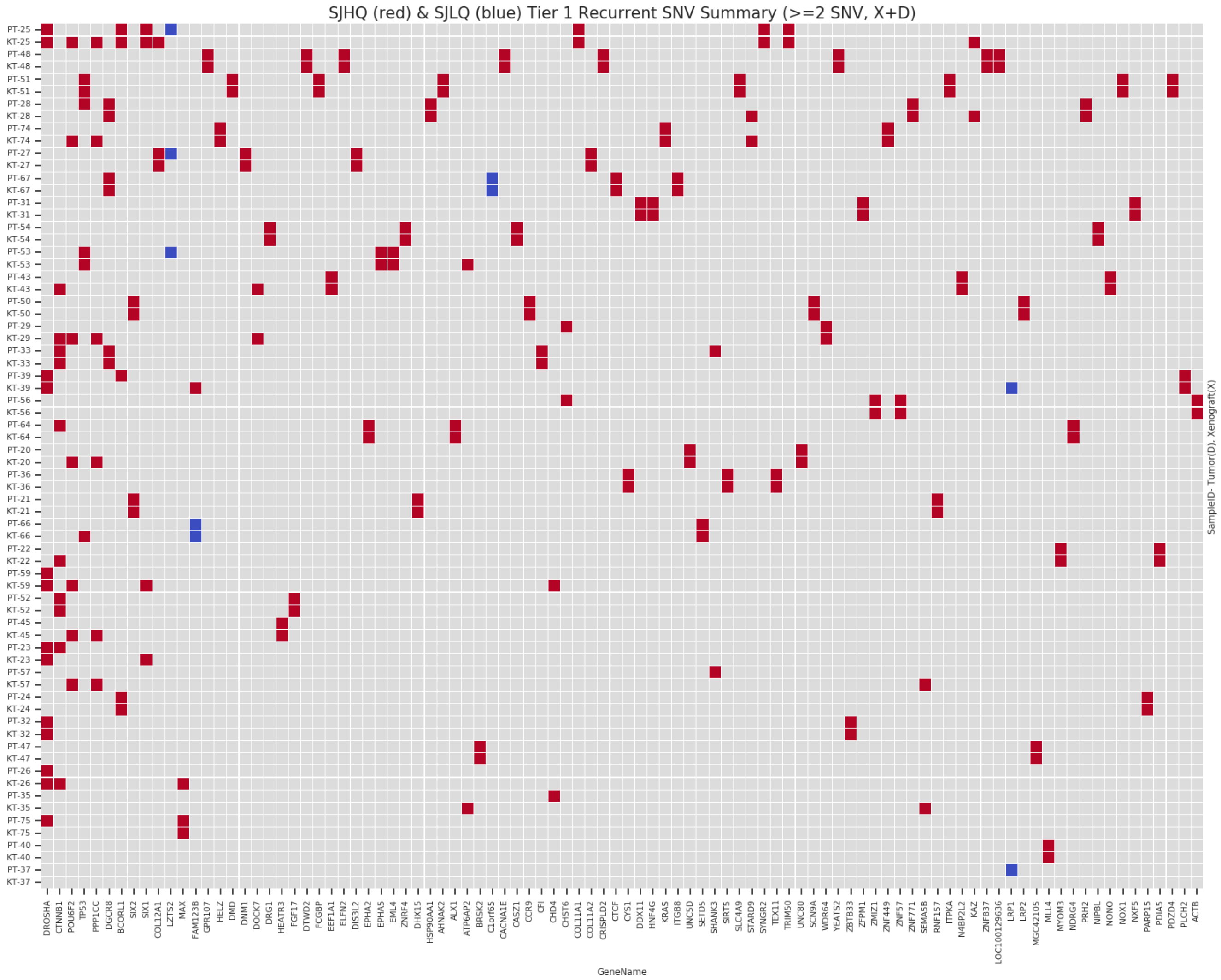

Supplementary Figure 3. Plot of somatic single nucleotide variants determined by whole exome sequencing. This plot includes genes for which variants were detected in at least two separate specimens. SJHQ = high quality mutations; SJLQ = low quality mutations. High quality single nucleotide variants present in at least two specimens were validated by target capture sequencing. Low quality single nucleotide variants were excluded from further analysis. PT = primary tumors, KT = xenografts (WTPDX).

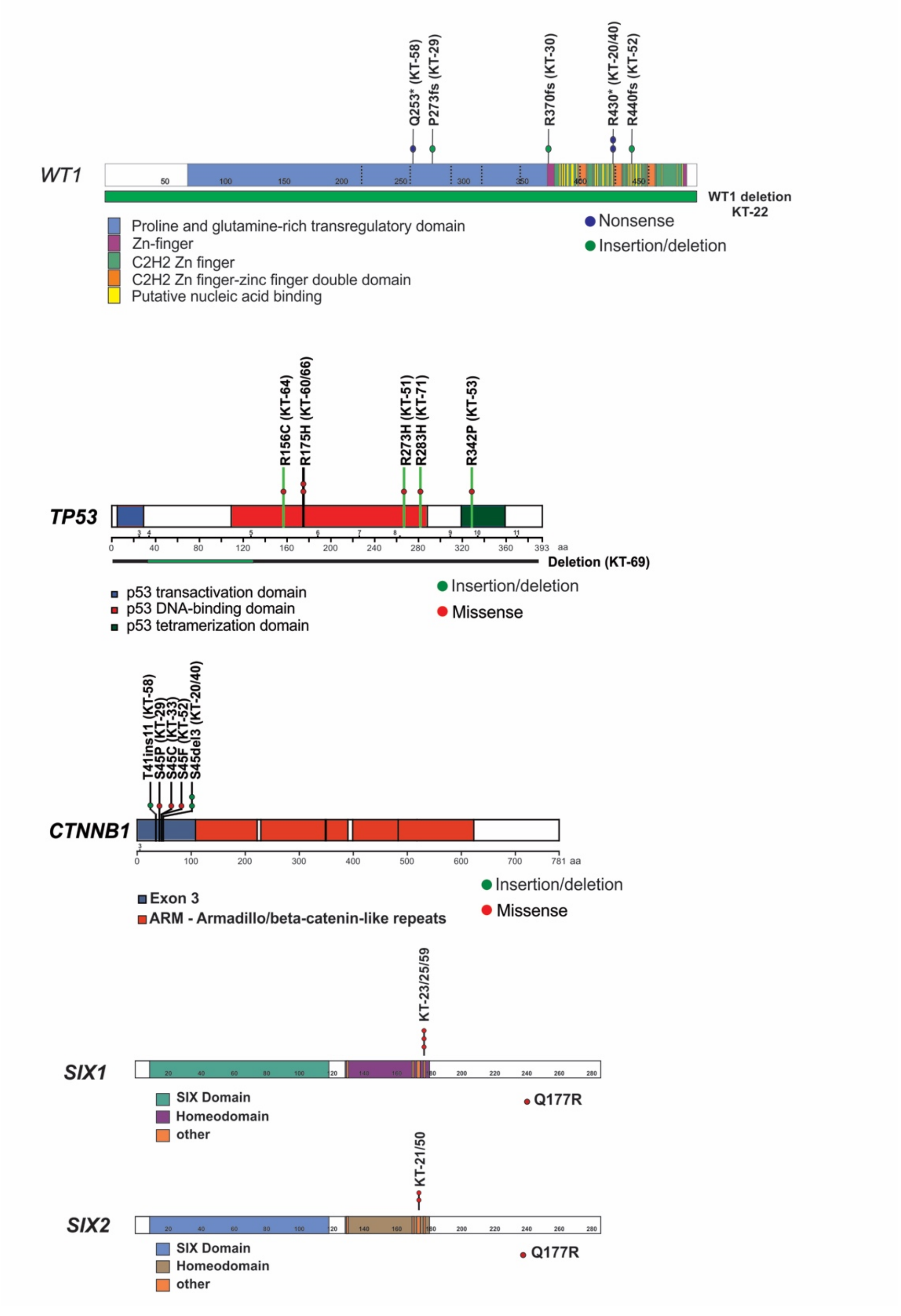

Supplementary Figure 4. Schematic diagram of *WT1*, *TP53*, *CTNNB1* exon 3, and *SIX1* and *SIX2* Q177R hotspot mutations detected in WTPDX by Sanger sequencing. The KT number of each xenograft containing the specified mutation is included.

A

Downregulated pathways (entire cohort)

Index	Name	P-value	Adjusted p-value	Z-score	Combined score
1	Integrin signalling pathway_Homo sapiens_P00034	0.00001535	0.0009824	-1.78	19.74
2	Cadherin signaling pathway_Homo sapiens_P00012	0.02401	0.7682	-1.51	5.62
3	PDGF signaling pathway_Homo sapiens_P00047	0.07342	0.9717	-1.46	3.81
4	Inflammation mediated by chemokine and cytokine signaling pathway_Homo sapiens_P00031	0.1311	0.9717	-1.32	2.69
5	Oxytocin receptor mediated signaling pathway_Homo sapiens_P04391	0.1186	0.9717	-1.22	2.61
6	Blood coagulation_Homo sapiens_P00011	0.1091	0.9717	-1.11	2.45
7	5HT2 type receptor mediated signaling pathway_Homo sapiens_P04374	0.1949	0.9717	-0.88	1.45
8	Endothelin signaling pathway_Homo sapiens_P00019	0.2389	0.9717	-0.74	1.06
9	FAS signaling pathway_Homo sapiens_P00020	0.1522	0.9717	-0.54	1.01
10	p38 MAPK pathway_Homo sapiens_P05918	0.1653	0.9717	-0.54	0.98

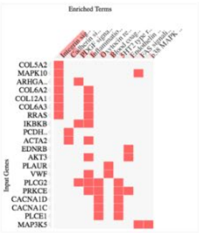

B

Upregulated pathways (entire cohort)

Index	Name	P-value	Adjusted p-value	Z-score	Combined score
1	De novo pyrimidine deoxyribonucleotide biosynthesis_Homo sapiens_P02739	0.01315	0.3835	-1.25	5.42
2	p53 pathway_Homo sapiens_P00059	0.03879	0.3835	-1.33	4.32
3	Parkinson disease_Homo sapiens_P00049	0.05794	0.3835	-1.23	3.49
4	Huntington disease_Homo sapiens_P00029	0.07293	0.3835	-1.27	3.32
5	Cell cycle_Homo sapiens_P00013	0.03266	0.3835	-0.95	3.24
6	Cytoskeletal regulation by Rho GTPase_Homo sapiens_P00016	0.1312	0.4518	-0.61	1.25
7	De novo purine biosynthesis_Homo sapiens_P02738	0.07874	0.3835	-0.46	1.16
8	p53 pathway feedback loops 2_Homo sapiens_P04398	0.1933	0.4609	-0.20	0.33
9	Ubiquitin proteasome pathway_Homo sapiens_P00060	0.1803	0.4609	-0.10	0.17
10	Wnt signaling pathway_Homo sapiens_P00057	0.9937	0.9937	1.11	-0.01

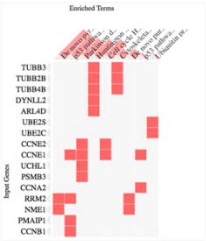

C

Downregulated pathways (lowest Spearman quartile)

Index	Name	P-value	Adjusted p-value	Z-score	Combined score
1	PDGF signaling pathway_Homo sapiens_P00047	0.000002023	0.0001537	-1.65	21.62
2	Integrin signalling pathway_Homo sapiens_P00034	0.0003273	0.01244	-1.69	13.53
3	FAS signaling pathway_Homo sapiens_P00020	0.008457	0.1505	-1.00	4.77
4	B cell activation_Homo sapiens_P00010	0.01776	0.2094	-1.15	4.63
5	p38 MAPK pathway_Homo sapiens_P05918	0.009903	0.1505	-0.97	4.50
6	Ras Pathway_Homo sapiens_P04393	0.01929	0.2094	-1.05	4.16
7	CCKR signaling map ST_Homo sapiens_P06959	0.03389	0.2771	-1.04	3.52
8	Interleukin signaling pathway_Homo sapiens_P00036	0.02921	0.2771	-0.94	3.31
9	JAK/STAT signaling pathway_Homo sapiens_P00038	0.007401	0.1505	-0.67	3.28
10	Apoptosis signaling pathway_Homo sapiens_P00006	0.03722	0.2771	-0.78	2.56

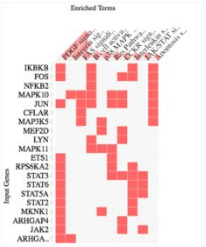

D

Upregulated pathways (lowest Spearman quartile)

Index	Name	P-value	Adjusted p-value	Z-score	Combined score
1	Cell cycle_Homo sapiens_P00013	0.0001038	0.005914	-1.28	11.76
2	Ubiquitin proteasome pathway_Homo sapiens_P00060	0.0003233	0.009213	-1.32	10.59
3	Parkinson disease_Homo sapiens_P00049	0.001004	0.01888	-1.33	9.16
4	Transcription regulation by bZIP transcription factor_Homo sapiens_P00055	0.02131	0.1563	-0.81	3.12
5	Huntington disease_Homo sapiens_P00029	0.02948	0.1680	-0.78	2.77
6	Glycolysis_Homo sapiens_P00024	0.01031	0.09797	-0.12	0.54
7	Integrin signalling pathway_Homo sapiens_P00034	0.9979	0.9979	3.57	-0.01
8	Inflammation mediated by chemokine and cytokine signaling pathway_Homo sapiens_P00031	0.9975	0.9979	3.70	-0.01
9	Cadherin signaling pathway_Homo sapiens_P00012	0.9972	0.9979	3.38	-0.01
10	Wnt signaling pathway_Homo sapiens_P00057	0.9970	0.9979	3.32	-0.01

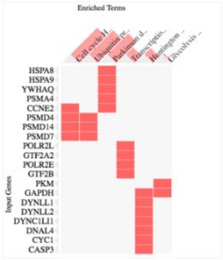

Supplementary Figure 5. (A) In the complete cohort, the integrin signaling pathway was noted to be significantly downregulated in WTPDX. (B) In the complete cohort, there were no statistically significant gene pathways upregulated in WTPDX When analysis was limited to the 9-primary tumor-WTPDX pairs that constituted the lowest quartile for Spearman correlation, (C) PDGF and integrin signaling pathways were found to be downregulated and (D) cell cycle and ubiquitin proteasome pathways were found to be upregulated in WTPDX. *P* values were calculated using Fisher exact test, adjusted *p* values were calculated using the Benjamini-Hochberg method for correction for multiple hypotheses testing.

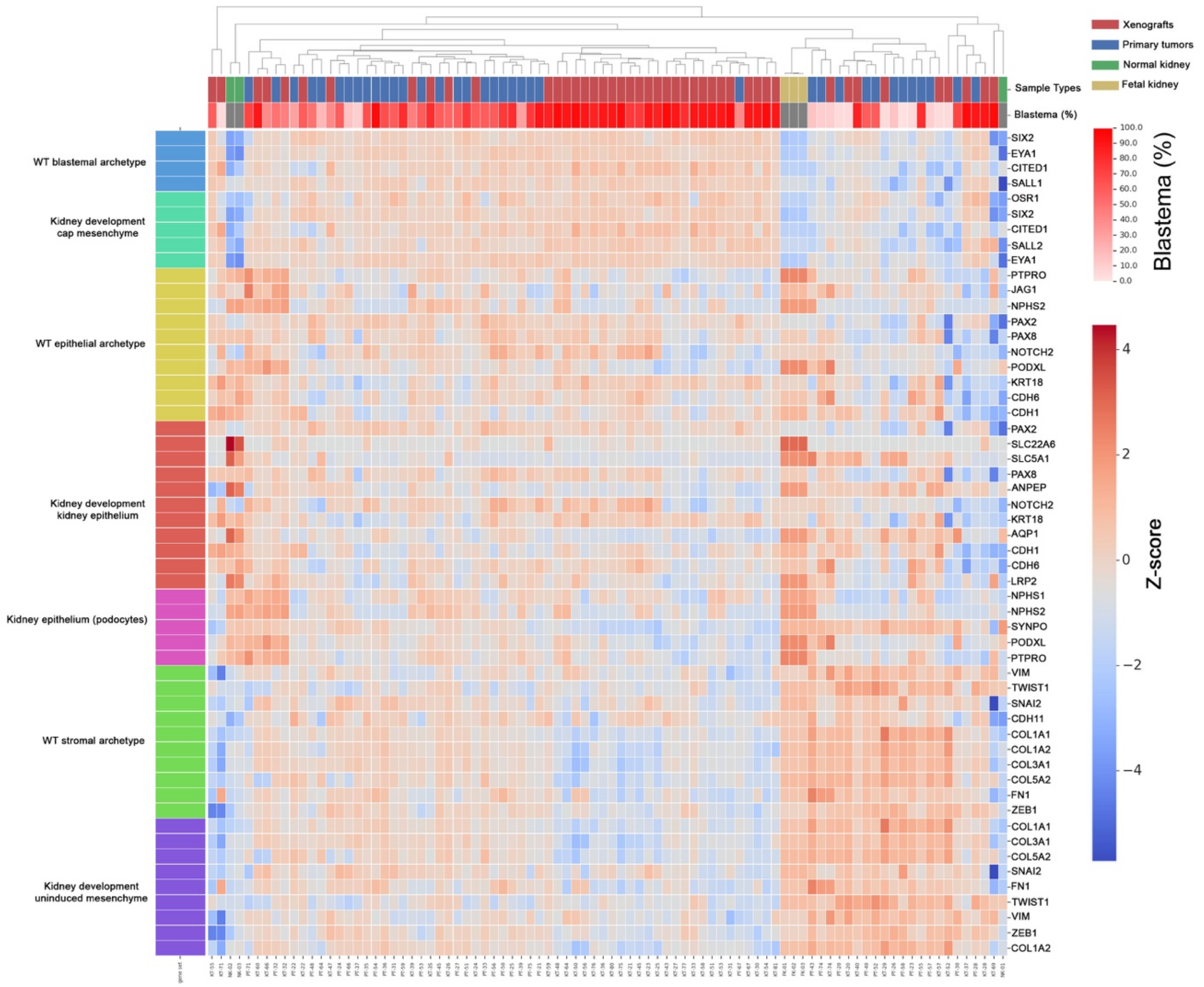

Supplementary Figure 6. Expression of WT histologic archetype and kidney developmental gene sets in primary tumors and WTPDX by RNA-seq. Primary tumors (PT), WTPDX (KT), adult normal kidney (NK) and pooled human fetal kidney RNA (FK) are clustered (columns) according to expression of WT histologic archetype gene sets (blastemal, epithelial, stromal) and gene sets associated with kidney developmental cellular lineages (cap mesenchyme, kidney epithelium, podocytes, uninduced mesenchyme). The majority of PT and KT show enrichment of cap mesenchyme and WT blastemal archetype genes when compared to normal and fetal kidney. Both PT and KT with low percentage of blastema by histology demonstrate reduced expression for cap mesenchyme and blastemal genes, but upregulation of stromal and uninduced mesenchyme genes. KT – xenografts, PT – primary tumor, NK – normal kidney, FK – pooled fetal kidney RNA. Heatmap = gene expression *z* scores.

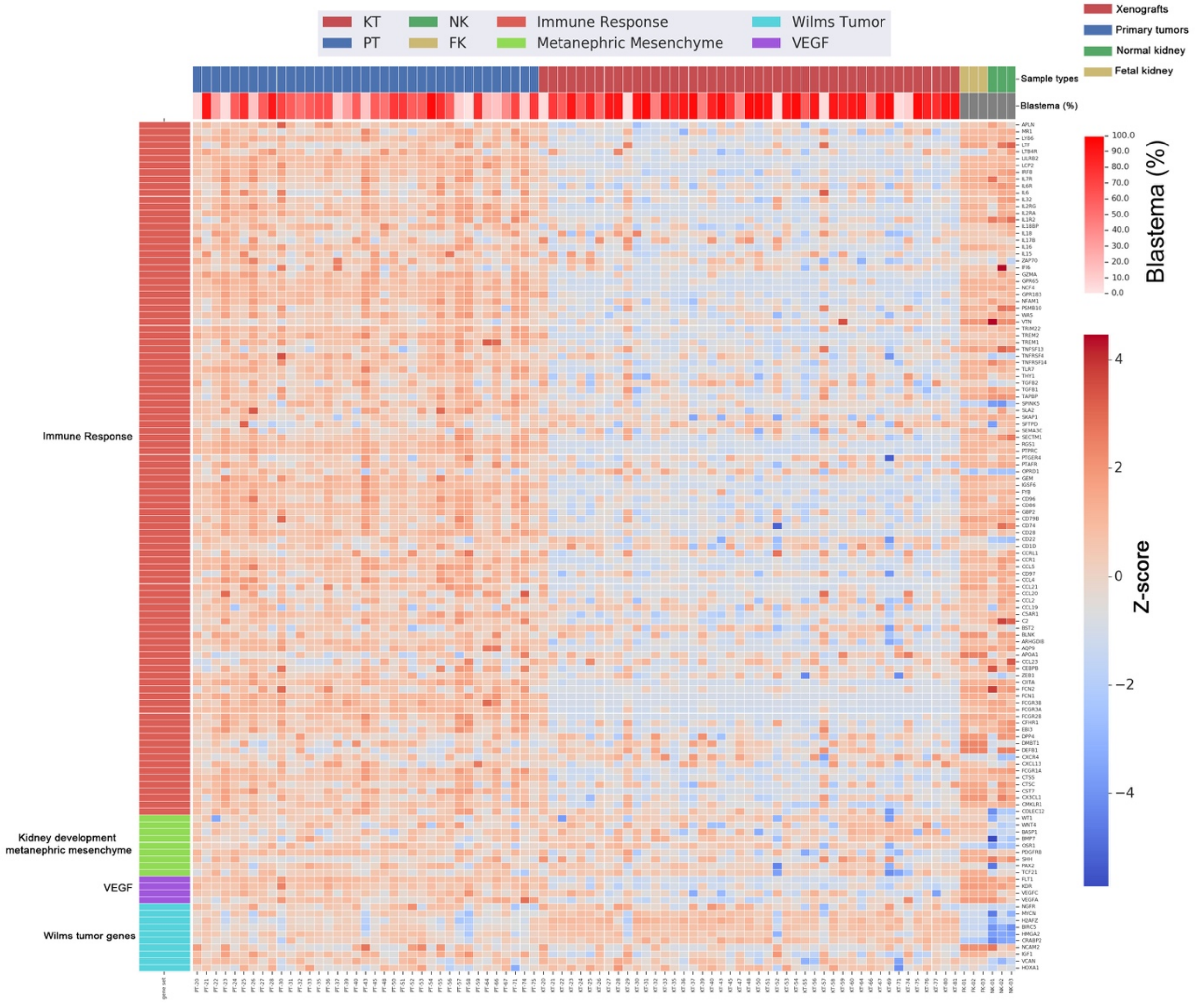

Supplementary Figure 7. WTPDX maintain gene expression of Wilms tumor and metanephric mesenchyme gene sets, but show decreased expression of gene sets associated with the immune response and VEGF by RNA-seq. This is a heatmap display of *z* score normalized relative gene expression using RNA-seq data. The percent blastema for each primary tumor (left portion of heatmap) and corresponding WTPDX (KT, right portion of heatmap) is displayed for reference. KT = xenografts/WTPDX, PT = primary tumors, NK = adult normal kidney specimens, FK = pooled human fetal kidney RNA. Heatmap = gene expression *z* scores.

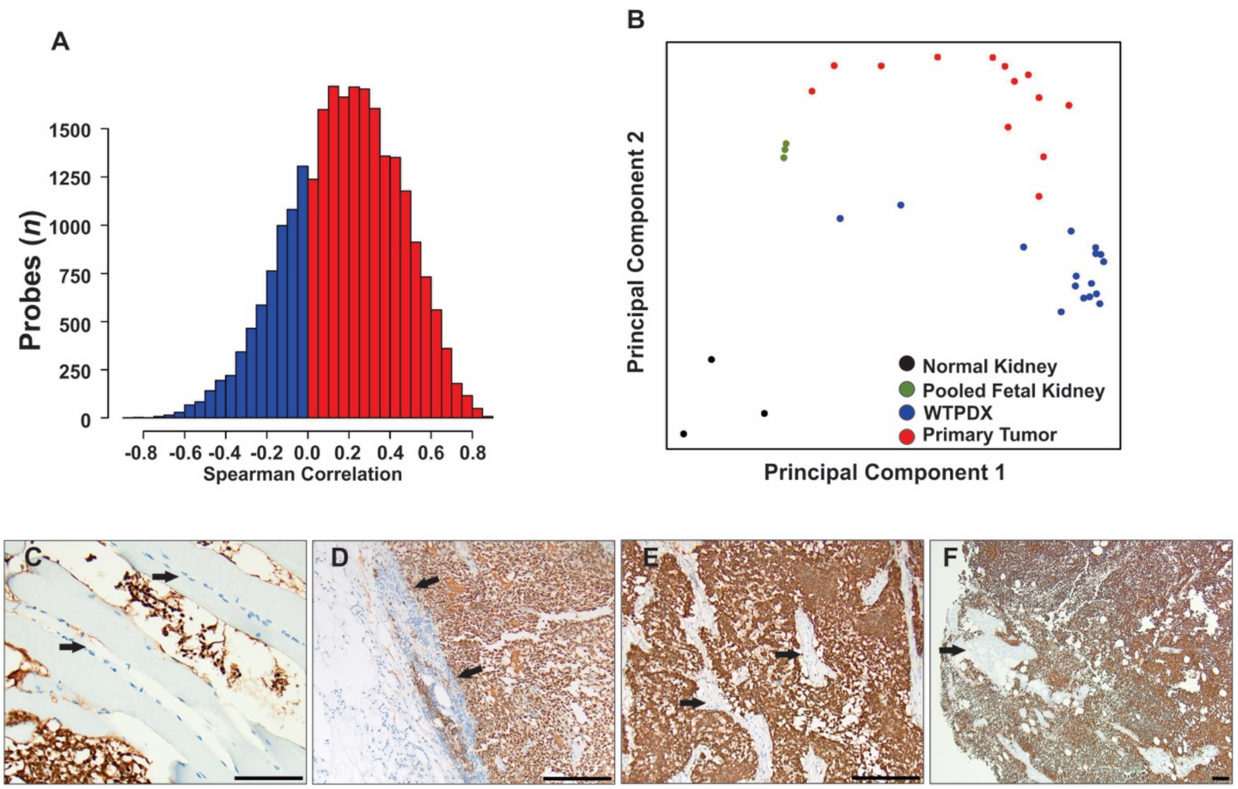

Supplementary Figure 8. Comparison of gene expression in WTPDX and primary tumors by RNA microarray. A selection of WTPDX analyzed by RNA microarray confirmed the RNA-seq findings. (A) Dominance of positively correlated gene expression (red) between primary tumors and WTPDX over negatively correlated gene expression (blue) indicates that WTPDX retained the transcriptome profiles of their originating primary tumors (B) Principal component analysis shows clustering of WTPDX (blue) with respect to primary WT (red), fetal kidney (green), and normal kidney (black) specimens. (C) NUMA1 staining demonstrates murine-derived tumor vasculature (negative endothelial cells; arrows) interspersed with human-derived immunopositive tumor cells. (D) Murine- derived tumor stroma with adjacent murine subcutaneous fat constitutes the capsular surface (arrows) of a WTPDX. (E) Intervening murine-derived tumor stroma (negative areas; arrows) within human-derived blastemal and epithelial tumor cells. (F) Human tumor cells (positive) are more predominant than murine- derived stroma (negative; arrow) in WTPDX. Scale bars = 100 µm.

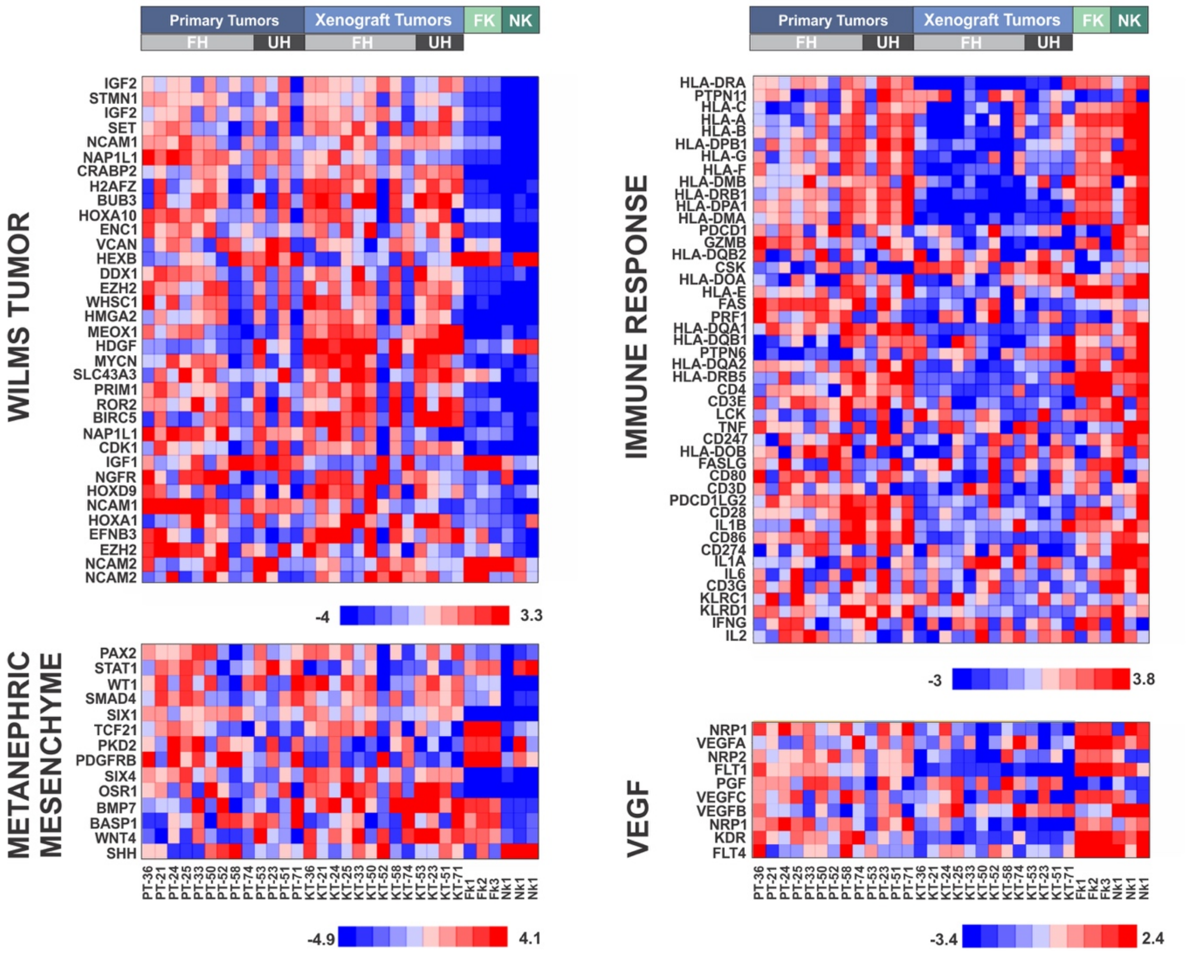

Supplementary Figure 9. Comparison of gene expression in primary tumors in WTPDX, fetal, and normal kidney specimens by RNA array. A selection of WTPDX analyzed by RNA microarray confirmed the RNA- seq findings shown in Supplementary Figure 6. WTPDX maintained expression of a WT gene set that included genes previously shown to be upregulated in WT compared with fetal and normal kidney (left upper panel). WTPDX also maintained expression of kidney developmental genes, with high expression of genes associated with the metanephric mesenchyme of the fetal kidney (lower left panel). WTPDX had decreased expression of immune response (upper right panel) and VEGF-related genes (lower right panel). FK, fetal kidney; NK, normal kidney; FH, favorable histology; UH, unfavorable histology. Heatmap values = gene expression *z* scores.

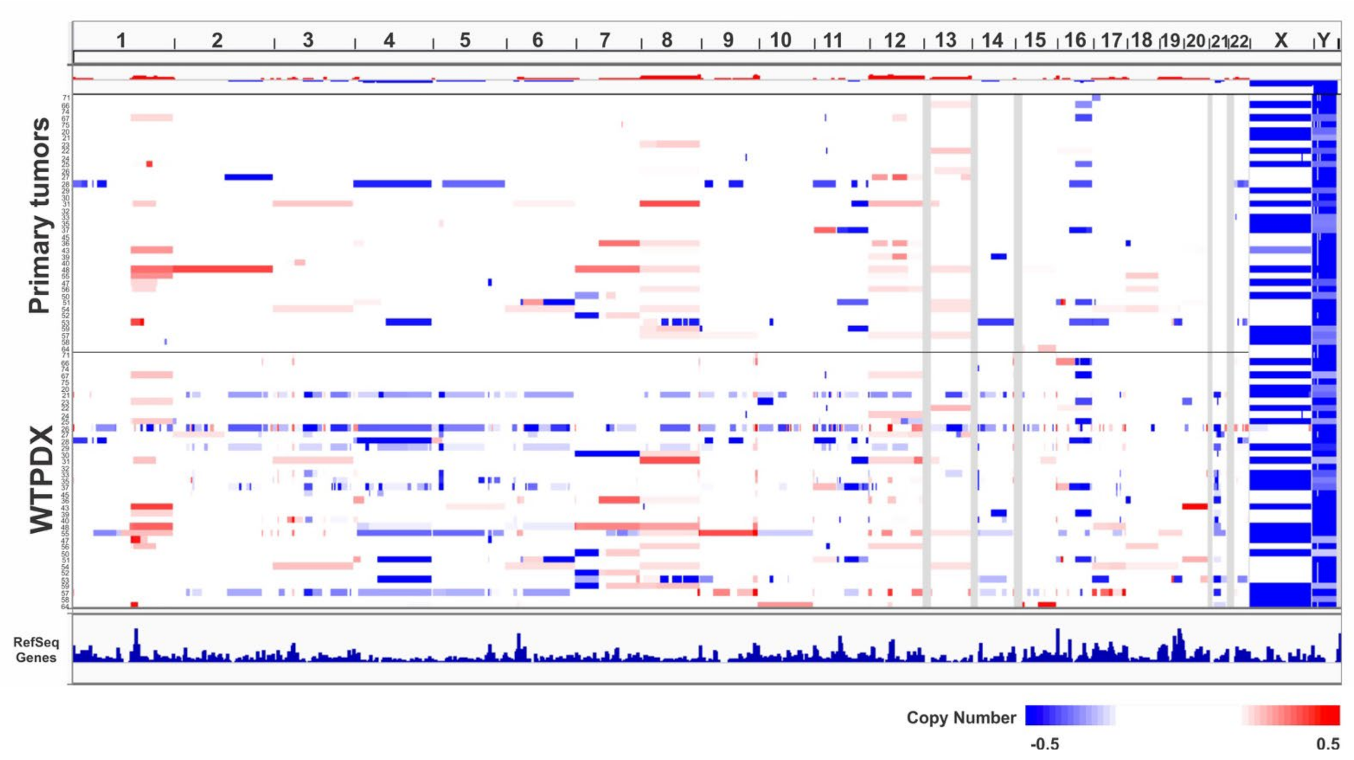

Supplementary Figure 10. Comparative chromosomal copy number analysis. Comparison of genome-wide chromosomal copy number alterations as determined by data from the Infinium MethylationEPIC BeadChip (850K) system in primary tumors (top panel) and WTPDX (bottom panel). Chromosomal copy number gains (red) and losses (blue) are displayed.

Supplementary References

1 Ma, X. *et al.* Pan-cancer genome and transcriptome analyses of 1,699 paediatric leukaemias and solid tumours. *Nature* **555**, 371-376, doi:10.1038/nature25795 (2018).

2 Miller, C. A. *et al.* Visualizing tumor evolution with the fishplot package for R. *BMC Genomics* **17**, 880, doi:10.1186/s12864-016-3195-z (2016).

3 Chen, E. Y. *et al.* Enrichr: interactive and collaborative HTML5 gene list enrichment analysis tool. *BMC Bioinformatics* **14**, 128, doi:10.1186/1471-2105-14-128 (2013).

4 Kuleshov, M. V. *et al.* Enrichr: a comprehensive gene set enrichment analysis web server 2016 update. *Nucleic Acids Res* **44**, W90-97, doi:10.1093/nar/gkw377 (2016).

5 Irizarry, R. A. *et al.* Exploration, normalization, and summaries of high density oligonucleotide array probe level data. *Biostatistics* **4**, 249-264, doi:10.1093/biostatistics/4.2.249 (2003).

6 Pounds, S. & Cheng, C. Robust estimation of the false discovery rate. *Bioinformatics* **22**, 1979-1987, doi:10.1093/bioinformatics/btl328 (2006).
