# Supplementary material for: Forty-five patient-derived xenografts capture the clinical and biological heterogeneity of Wilms tumor
Source: Nat Commun. 2019 Dec 20;10:5806. doi: 10.1038/s41467-019-13646-9 (PMC6925259; doi:10.1038/s41467-019-13646-9)
Supplement: Supplementary file 3 — Description of Additional Supplementary Files [file 41467_2019_13646_MOESM3_ESM.docx]

**Description of Supplementary Files**

**File Name:** Supplementary Data 1

**Description:** Short tandem repeat profiling of Wilms tumors

**File Name:** Supplementary Data 2

**Description:** Histology comparison and patient treatments

**File Name:** Supplementary Data 3

**Description:** Whole exome sequencing summary data

**File Name:** Supplementary Data 4

**Description:** RNA-seq summary data

**File Name:** Supplementary Data 5

**Description:** Gene expression microarray summary data

**File Name:** Supplementary Data 6

**Description:** PCR primers for Sanger sequencing
