# Supplementary material for: Forty-five patient-derived xenografts capture the clinical and biological heterogeneity of Wilms tumor
Source: Nat Commun. 2019 Dec 20;10:5806. doi: 10.1038/s41467-019-13646-9 (PMC6925259; doi:10.1038/s41467-019-13646-9)
Supplement: Supplementary file 10 — Reporting Summary [file 41467_2019_13646_MOESM10_ESM.pdf]

nature research

Corresponding author(s): Andrew J Murphy

Reporting Summary

Nature Research wishes to improve the reproducibility of the work that we publish. This form provides structure for consistency and transparency in reporting. For further information on Nature Research policies, see Authors & Referees and the Editorial Policy Checklist.

Statistical parameters

When statistical analyses are reported, confirm that the following items are present in the relevant location (e.g. figure legend, table legend, main text, or Methods section).

n/a	Confirmed	
☐	☒	The exact sample size (*n*) for each experimental group/condition, given as a discrete number and unit of measurement
☐	☒	An indication of whether measurements were taken from distinct samples or whether the same sample was measured repeatedly
☐	☒	The statistical test(s) used AND whether they are one- or two-sided<br>*Only common tests should be described solely by name; describe more complex techniques in the Methods section.*
☐	☒	A description of all covariates tested
☐	☒	A description of any assumptions or corrections, such as tests of normality and adjustment for multiple comparisons
☐	☒	A full description of the statistics including central tendency (e.g. means) or other basic estimates (e.g. regression coefficient) AND variation (e.g. standard deviation) or associated estimates of uncertainty (e.g. confidence intervals)
☐	☒	For null hypothesis testing, the test statistic (e.g. *F*, *t*, *r*) with confidence intervals, effect sizes, degrees of freedom and *P* value noted<br>*Give P values as exact values whenever suitable.*
☒	☐	For Bayesian analysis, information on the choice of priors and Markov chain Monte Carlo settings
☒	☐	For hierarchical and complex designs, identification of the appropriate level for tests and full reporting of outcomes
☒	☐	Estimates of effect sizes (e.g. Cohen's *d*, Pearson's *r*), indicating how they were calculated
☐	☒	Clearly defined error bars<br>*State explicitly what error bars represent (e.g. SD, SE, CI)*

Our web collection on statistics for biologists may be useful.

Software and code

Policy information about availability of computer code

Data collection

See below for software used. The software used for data collection was also used for some level of data analysis.

Data analysis

Adobe Photoshop v13.0 (13.0 20120315.r.428 2012/03/15:21:00:00) x64 (Adobe, San Jose, CA)
Affymetrix Expression Console software (ThermoFisher Scientific, Waltham, MA)
Coffalyser.Net v.140721.1958 (MRC Holland, Amsterdam, The Netherlands)
Coreldraw X6 (64-Bit) (Corel Corporation, Ottawa, Canada)
GENCODE v19 (https://www.gencodegenes.org/)
GenomeStudio Methylation Module v1.8 (Illumina, San Diego, CA)
GeneMapper Software v5 (ThermoFisher Scientific, Waltham, MA)
GraphPad Prism v7.0d (GraphPad Software, La Jolla, CA)
Integrated Genomics Viewer (IGV) v2.4.8 (Broad Institute, Cambridge, MA)
Microsoft Excel 2016 MSO(16.0.8431.2110) 32-bit (Microsoft, Redmond, WA)
Microsoft Word 2016 MSO(16.0.8431.2110) 32-bit (Microsoft, Redmond, WA)
Olympus cellSens Standard v1.18 (Build 16686) (Olympus, Tokyo, Japan)
Protein Analysis Through Evolutionary Relationships (PANTHER) database v13.1 (http://www.pantherdb.org/)
Enrichr tool 2016 update (https://amp.pharm.mssm.edu/Enrichr/)
Python v2.7.2 (https://www.python.org/download/releases/2.7.2/) Python Software Foundation, Wilmington, DE) using the following

packages:
--SciPy (https://www.scipy.org/)
--Pandas (https://pandas.pydata.org/)
--NumPy (http://www.numpy.org/)
--Scikit-learn (https://scikit-learn.org/stable/)
--Seaborn (https://seaborn.pydata.org/)
R Software v3.5.1 (www.rproject.org) using the following packages:
--DNAcopy Bioconductor package v1.56.0 (https://bioconductor.org/packages/release/bioc/html/DNAcopy.html)
--HTseq-count v0.8.0 (https://htseq.readthedocs.io/en/master/count.html)
--minifi Bioconductor package v1.28.0 (https://bioconductor.org/packages/release/bioc/html/minfi.html)
--fishplot (https://www.ncbi.nlm.nih.gov/pubmed/27821060)
Sequence Scanner Software v2.0 (Applied Biosystems, Foster City, CA)

For manuscripts utilizing custom algorithms or software that are central to the research but not yet described in published literature, software must be made available to editors/reviewers upon request. We strongly encourage code deposition in a community repository (e.g. GitHub). See the Nature Research guidelines for submitting code & software for further information.

Data

Policy information about availability of data

All manuscripts must include a data availability statement. This statement should provide the following information, where applicable:

- Accession codes, unique identifiers, or web links for publicly available datasets
- A list of figures that have associated raw data
- A description of any restrictions on data availability

Whole exome sequencing and RNA-seq data are available in the European Genome-phenome Archive (EGA) database (https://www.ebi.ac.uk/ega/home) under accession number EGAS00001003361.The RNA expression data and methylation profiling data that support the findings of this study are available in the Gene Expression Omnibus (GEO) database (https://www.ncbi.nlm.nih.gov/geo/) under identifiers GSE110696 (gene expression) and GSE110697 (methylation). All Wilms tumor patient derived xenografts used in this study are available to the scientific community upon request.

Field-specific reporting

Please select the best fit for your research. If you are not sure, read the appropriate sections before making your selection.

[x] Life sciences   [ ] Behavioural & social sciences   [ ] Ecological, evolutionary & environmental sciences

For a reference copy of the document with all sections, see nature.com/authors/policies/ReportingSummary-flat.pdf

Life sciences study design

All studies must disclose on these points even when the disclosure is negative.

Sample size	A sample size calculation was not performed. The sample size was determined by the number of available Wilms tumor patient-derived xenografts that were established between 2007 and 2016. The number of corresponding germline DNA and primary tumor tissues was determined by biobanked specimens with appropriate consent for genomic studies.
Data exclusions	We attempted to establish heterotopic Wilms tumor patient-derived xenografts from 83 patient tumor samples following resection performed at St. Jude Children's Research Hospital from 2007 to 2016. Of those, 64 were successfully engrafted (engraftment rate 77.1%). 17 initially engrafted xenograft models were lost due to a technical storage issue (freezer failure). This left 47 potential xenografts to include in this study. Two xenografts were subsequently excluded because short tandem repeat (STR) DNA profiling could not detect human DNA. This left 45 xenografts for molecular analysis.<br><br>45 total xenografts were available for all molecular analyses (with the exception of RNA-seq and whole exome sequencing)<br>39 total primary tumors were available for molecular analyses (with the exception of RNA-seq and whole exome sequencing)<br>37 primary tumor-xenograft pairs were available for RNA-seq after RNA quality control analysis for RNA-sequencing eliminated 2 specimens.<br>35 germline-primary tumor-xenograft DNA trios were available for whole exome sequencing<br>35 germline-primary tumor-xenograft DNA trios were available for Target capture sequencing validation
Replication	Whole exome sequencing findings were confirmed by target-capture sequencing of all variants and focused Sanger sequencing for genes and hotspots of interest (TP53, WT1, CTNNB1 exon 3, SIX1 Q177R hotspot, SIX2 Q177R hotspot, N-MYC P44L hotspot). Loss of heterozygosity at 11p15 by MLPA was confirmed by custom genotyping of a panel of microsatellite markers at 11p15 (D11S1363, D11S922, D11S4046, HUMTH01, and D11S988). RNA-sequencing findings were corroborated by expression microarray analysis.
Randomization	Nothing to disclose. This is not an intervention study, so randomization is not applicable.

Blinding	Nothing to disclose. This is not an intervention study, so randomization is not applicable.

Reporting for specific materials, systems and methods

Materials & experimental systems

n/a	Involved in the study
	☒ Unique biological materials
	☒ Antibodies
☒	☐ Eukaryotic cell lines
☒	☐ Palaeontology
	☒ Animals and other organisms
	☒ Human research participants

Methods

n/a	Involved in the study
☒	☐ ChIP-seq
☒	☐ Flow cytometry
☒	☐ MRI-based neuroimaging

Unique biological materials

Policy information about availability of materials

Obtaining unique materials	The heterotopic xenografts detailed in this manuscript are a unique biological material and are freely available to the scientific community upon request.

Antibodies

Antibodies used	Immunohistochemistry:<br>1. anti-human p53 antibody (clone name DO-7); Zeta Corp catalog # Z2029M (Mouse monoclonal, 1:200 dilution)<br>2. anti-human WT1 antibody (clone name 6F-H2); Dako catalog # M3561 (Mouse monoclonal, 1:25 dilution)<br>3. anti-human SIX2 antibody; Proteintech catalog # 11562-1-AP (Rabbit polyclonal, 1:50 dilution)<br>4. anti-human NUMA1 antibody; Lifespan Biosciences catalog # LS-B11047 (Rabbit polyclonal, 1:75 dilution)<br>5. Anti-mouse CD3-epsilon antibody (clone name M-20); Santa Cruz Biotechnology catalog # sc-1127 (Goat polyclonal, 1:1000 dilution)
Validation	Antibodies were validated for use in these systems by the manufacturers who have provided references on each of their websites using the catalog numbers provided above.<br><br>Zeta Corp - http://zeta-corp.com/<br>Dako - https://www.labome.com/product/Dako/M3561.html<br>Proteintech - https://www.ptglab.com/products/SIX2-Antibody-11562-1-AP.htm<br>Lifespan Biosciences - https://www.lsbio.com/antibodies/numa1-antibody-numa-antibody-aa900-950-if-immunofluorescence-ihc-ihc-plus-ls-b11047/302082<br>Santa Cruz Biotechnology - https://www.scbt.com/scbt/product/cd3-epsilon-antibody-m-20

Animals and other organisms

Policy information about studies involving animals; ARRIVE guidelines recommended for reporting animal research

Laboratory animals	Male CB17 scid-/- mice (model # CB17SC-M; strain nomenclature - C.B-Igh-1b/IcrTac-Prkdcscid) between 6-8 weeks old were obtained from Taconic Farms, Hudson, NY and used for heterotopic xenografting studies.
Wild animals	The study did not involve wild animals.
Field-collected samples	Tissues from freshly resected male and female human Wilms tumors were expeditiously transported from the operating room to the pathology lab in RPMI at room temperature. Samples for research purposes were temporarily stored in RPMI at 4 degrees. Tissues from these human primary Wilms tumors were then snap frozen in liquid nitrogen for biobanking or heterotopically transplanted into the flanks of male CB17 scid -/- mice for xenografting. Samples were also fixed in 10% neutral buffered formalin and subsequently embedded into paraffin blocks for subsequent histologic and immunohistochemical studies.

Human research participants

Policy information about studies involving human research participants

Population characteristics	This study did not actually involve human subjects directly. We collected banked, de-identified tumor samples that were previously collected from patients <18 years of age.
Recruitment	Patients were not recruited into the study. Only banked, de-identified patient samples were used for this study.
